# Utilization of native oxygen in Eu(RE)-doped GaN for enabling device compatibility in optoelectronic applications

**DOI:** 10.1038/srep18808

**Published:** 2016-01-04

**Authors:** B. Mitchell, D. Timmerman, J. Poplawsky, W. Zhu, D. Lee, R. Wakamatsu, J. Takatsu, M. Matsuda, W. Guo, K. Lorenz, E. Alves, A. Koizumi, V. Dierolf, Y. Fujiwara

**Affiliations:** 1 Department of Physics and Astronomy, University of Mount. Union, 1972 Clark Ave, Alliance, OH, 44601, USA; 2 Division of Materials and Manufacturing Science, Graduate School of Engineering, Osaka University, Suita, Osaka 565-0871, Japan; 3 Center for Nanophase Materials Sciences, Oak Ridge National Laboratory, Oak Ridge, TN 37831, USA; 4 Instituto Superior Técnico, Campus Tecnológico e Nuclear, Estrada Nacional 10, P-2695-066 Bobadela LRS, Portugal; 5 Department of Physics and Astronomy, Lehigh University, 16 Memorial Dr. E, Bethlehem, PA, 18015, USA

## Abstract

The detrimental influence of oxygen on the performance and reliability of V/III nitride based devices is well known. However, the influence of oxygen on the nature of the incorporation of other co-dopants, such as rare earth ions, has been largely overlooked in GaN. Here, we report the first comprehensive study of the critical role that oxygen has on Eu in GaN, as well as atomic scale observation of diffusion and local concentration of both atoms in the crystal lattice. We find that oxygen plays an integral role in the location, stability, and local defect structure around the Eu ions that were doped into the GaN host. Although the availability of oxygen is essential for these properties, it renders the material incompatible with GaN-based devices. However, the utilization of the normally occurring oxygen in GaN is promoted through structural manipulation, reducing its concentration by 2 orders of magnitude, while maintaining both the material quality and the favorable optical properties of the Eu ions. These findings open the way for full integration of RE dopants for optoelectronic functionalities in the existing GaN platform.

Gallium Nitride is a wide band gap semiconductor material that has received considerable interest due its favorable thermal, electrical, and optical properties that make it an ideal material for applications in solid state-lighting^1^,^2^,^3^,^4^. The fabrication of devices based on GaN is commonly performed by organo-metallic vapor phase epitaxy (OMVPE), which incorporates high levels (~10^17^ cm^−3^) of oxygen into the crystal structure. The success of high-powered blue LEDs made from InGaN/GaN multiple quantum well (MQW) structures is largely due to the control and reduction of defects such as oxygen. Oxygen is known to impede device performance and reliability^5^, and much effort has gone into studying this defect and modifying growth conditions to minimize its presence in GaN-based devices^6^,^7^. The presence of oxygen in GaN, regardless of concentration, is normally discussed with a purely negative connotation, where possible positive aspects of its influence are not considered. For the continued optimization of this material, the positive and negative roles of critical defects, such as oxygen, need to be explored.

While the interaction of oxygen with inherent defects in GaN such as gallium vacancies and dislocations has been extensively investigated^8^,^9^,^10^,^11^,^12^,^13^,^14^,^15^,^16^,^17^,^18^, its influence and compatibility with co-dopants such as rare earth (RE) ions, is not well understood. (RE) ions are commonly used as dopants in GaN due to their favorable photonic, optoelectronic and magnetic properties^19^,^20^,^21^,^22^,^23^,^24^. The addition of oxygen in RE doped Si and GaAs was found to be critical to its operation^25^,^26^,^27^,^28^,^29^, however, in studies on O and Er co-implanted GaN, some groups report a positive influence of adding oxygen^27^,^30^,^31^, while others reported no effect^32^,^33^ or even a negative impact^34^,^35^. While there is theoretical evidence that RE ions will form complexes with oxygen in GaN^36^, which could impact the material, this has never been experimentally verified.

Moreover, the role of O has not been studied in organo-metallic epitaxial grown GaN:RE, the most commonly used growth technique in industry. For this method, the most frequently used RE precursors also contain oxygen in their molecular structure, which is then incorporated into the material during growth. This can potentially lead to the same detrimental effects for devices that high ambient oxygen concentrations do, but this is often disregarded in studies^20^,^21^,^37^,^38^,^39^; however this issue needs to be addressed to realize the commercialization of these materials. In this report we demonstrate that, depending on the growth conditions, O can have a positive or negative influence on GaN:RE. Knowledge of the underlying mechanism responsible for this enabled the optimization of the material, limiting the incorporation of O.

From the available RE ions we will focus on Eu, which is especially interesting for integration with GaN-based LEDs due to their bright, sharp, and stable red emission^40^,^41^. GaN:Eu is a promising material for the active layer of a GaN-based red LED, which has garnered considerable attention over the last few years, with preliminary LED devices containing a single 300nm active layer having already been demonstrated^38^,^42^. As with other work on GaN:RE, the RE source (in this case Eu(DPM)_3_), contained significant concentrations of oxygen in its molecular structure. In a recent publication on GaN:Eu, the O concentration, as determined by secondary ion mass spectroscopy (SIMS), was found to be about two orders of magnitude higher (~10^19^ cm^−3^) than that of commercially available GaN-based devices^43^, which is not realistic for maximizing device performance and reliability. In order to truly understand the role of oxygen, a new oxygen-free Eu source was developed, EuCp^pm^_2_, which allowed full control of the O content by means of co-doping.

## Results

**Figure 1:**
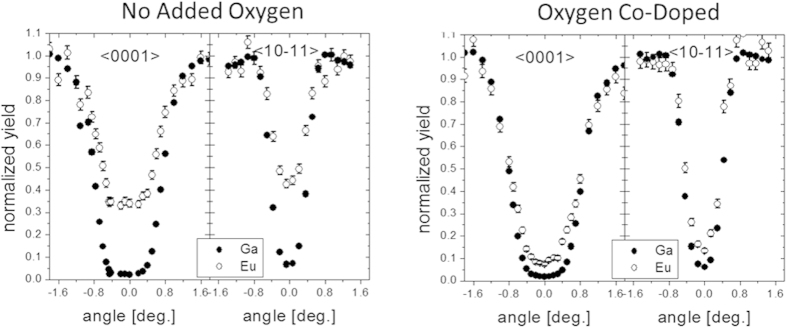
Rutherford Back Scattering results for GaN:Eu samples grown using EuCp^pm^_2_ without oxygen co-doping (left) and with oxygen co-doping (right). The sample with oxygen co-doping shows that almost every Eu ion sits on a Ga sites. Conversely, without oxygen only 70% of the Eu ions sit around Ga sites, the rest exist as interstitials.

To investigate the influence of oxygen on the structural properties of the Eu incorporation, Rutherford backscattering/channeling (RBS/C) measurements were performed (See Fig. 1). In conventional samples, as with all GaN:RE, the majority of the RE ions sit at Ga sites^36^,^44^,^45^. However, when the O was removed from the Eu source, it was found that only 70% of the Eu atoms were located on Ga sites, the majority of which were slightly displaced. Only when oxygen was reintroduced via co-doping was the normal RBS/C profile recovered. Additional details of the RBS/C results can be found in Supplementary Information Section 1.

More evidence of Eu instability without intentional addition of oxygen can be seen at the sample surfaces. When additional oxygen is not supplied during growth, Eu precipitation is observed on the samples surface. Such precipitation is not observed once intentional O_2_ is supplied, which increases the O content in the sample by an order of magnitude. Thus, without sufficient incorporation of O in the material, some of the Eu atoms are not incorporated, and diffuse to the surface. Along with the RBS results, it is clear that absence of oxygen has a detrimental effect on the nature of the Eu incorporation, and resulting structural properties of the material. It is therefore essential to also investigate the influence of oxygen on the optical properties of GaN:Eu. Additional figures and details regarding the surface precipitation can be found in Supplementary Information Section 2.

**Figure 2:**
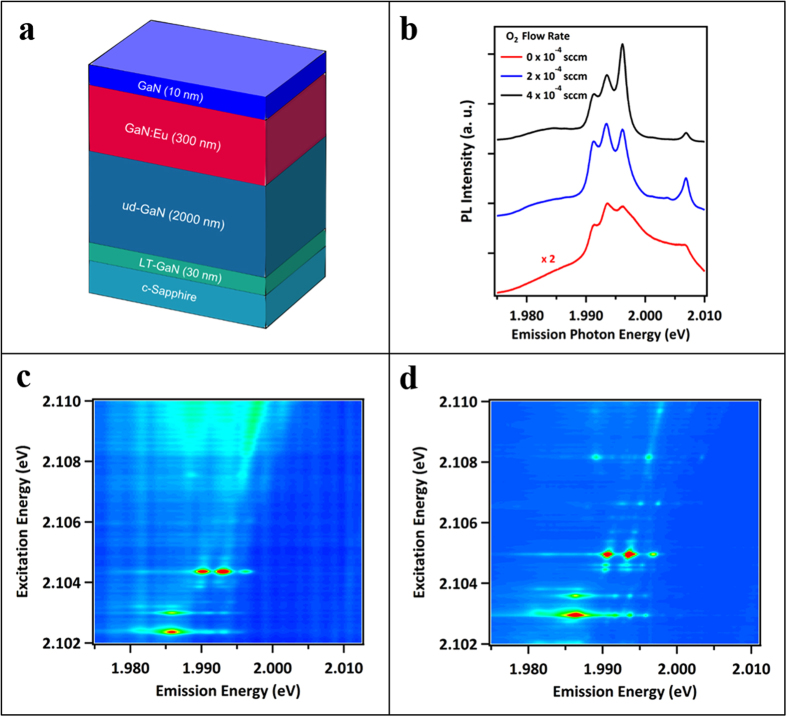
(**a**) Schematic of continuous growth (CG) sample structure. (**b**) The PL spectra under indirect excitation of GaN:Eu samples grown with fixed Eu source conditions, but various O flow rates. (**c**,**d**) represent CEES maps of the continuously grown samples with and without O co-doping, respectively.

The typical structure of the active layer is a 300 nm thick single layer of GaN:Eu grown on a GaN buffer and sapphire substrate, as shown in Fig. 2a. The Eu ions in the layer were excited indirectly, via electron hole pairs, to explore the emission quality and energy transfer efficiency of the samples, and resonantly in order to acquire information about the different emission centers and variation in local structures hereof (Combined Excitation Emission Spectroscopy, CEES, see Methods for details). For both excitation methods, the resulting photoluminescence spectra were found to be broad, with significant evidence for inhomogeneity in the local environments around the Eu ions, when low concentrations of oxygen were available (Fig. 2b,c). Furthermore, from the indirect excitation spectra it can be observed that the energy transfer efficiency was relatively poor. However, the emission spectra became increasingly sharp and uniform, and the energy transfer efficiency improved as oxygen was reintroduced with higher flow rates (Fig. 2b,d). It should be noted that when the oxygen flow rate was too high, the luminescence spectra became broad and distorted once again, indicating that oxygen can have a negative impact if it is overly supplied to the system. Additional figures and details of the photoluminescence (PL) results can be found in Supplementary Information Section 3.

Since the oxygen influences how Eu is incorporated into the crystal, it was considered that the converse may also be true. To explore this, four samples were grown with increasing Eu flow rates, but a fixed O_2_ flow rate. As expected, the Eu concentration increased with increasing Eu flow rate, however, the oxygen concentration also increased. Interestingly, the ratio of O to Eu remained relatively fixed at ~2.5%, for all Eu flow rates. This implies there is a link between the number of incorporated Eu and O atoms, as long as a constant source of O_2_ is supplied. When O_2_ was not intentionally flowed into the growth chamber with a Eu flow rate of 1.0 slm, the Eu and O concentrations were found to be ~4 × 10^19^ cm^−3^ and 2 × 10^17^ cm^−3^, respectively. The resulting O/Eu ratio is ~0.5%, which is almost an order of magnitude lower than when O_2_ is supplied. Details of the SIMS measurements can be found in Supplementary Information Section 4.

These results strongly indicate that for single layers of GaN:Eu, significant concentrations of oxygen are required to ensure uniform Eu incorporation and favorable optical properties. However, for the high performance and reliability of GaN-based devices, the minimization of oxygen is essential. It is clear that these two requirements are not mutually compatible, and it is this critical issue that we turn our attention to, and resolve, in the remainder of this article.

**Figure 3:**
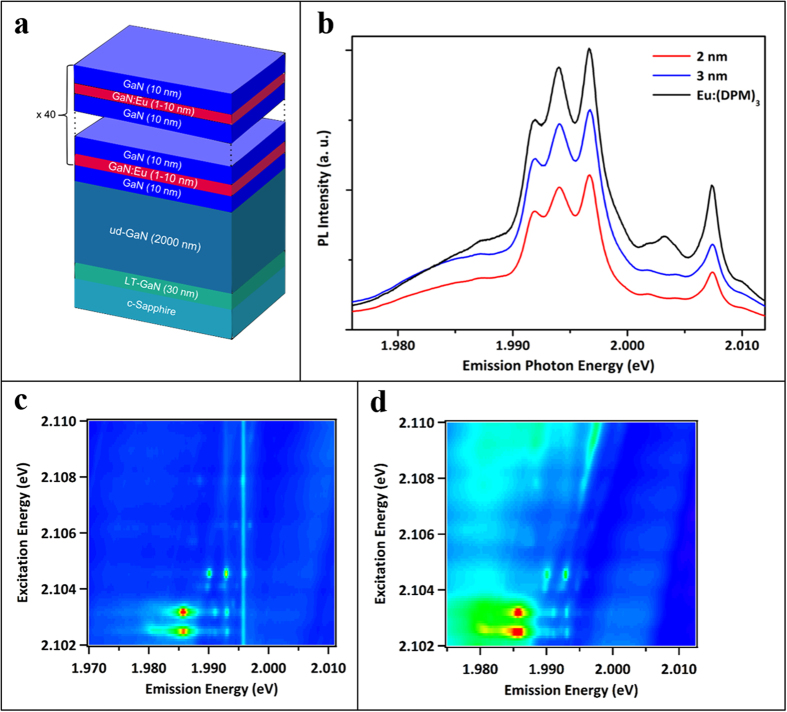
(**a**) Schematic of delta structure (DS) sample structure. (**b**) PL emission intensities of the most efficient Eu(DPM)_3_ sample compared with three DS samples of increasing Eu layer thickness. (**c**,**d**) represent CEES maps of the DS samples with GaN to GaN:Eu layer thickness ratios of 10:3 and 10:10, respectively.

The SIMS results mentioned above indicate that as the Eu concentration is reduced, the concentration of O necessary to stabilize its incorporation is also reduced. To optimize these devices and make them compatible with commercially available GaN devices, the O content in the sample needs to be reduced. In other words, these devices need to be fabricated without the flow of additional oxygen in the growth chamber. This has been a huge challenge because, as seen from the previous results, Eu incorporation into the host requires additional O for stabilization. To overcome this challenge, delta structure (DS) samples with alternating layers of Eu-doped and un-doped GaN, were grown without the intentional flow of O_2_. The un-doped GaN was held at a constant thickness of 10nm, while the GaN:Eu layers were varied from 1nm-10nm, without intentional O_2_ supplied during growth of either layer (see Fig. 3a for schematic of the structure).

The DS samples were excited above the bandgap to explore the effect of the GaN:Eu layering on the emission spectra and excitation efficiency. The PL emission spectra of two DS samples are shown in Fig. 3b, which is compared with the spectrum of the brightest (continuous growth; CG), oxygen containing Eu source (Eu:(DPM)_3_) sample. It is immediately apparent that despite the lack of additional oxygen, the emission spectra of the DS samples are very sharp.

Furthermore, it is expected that the emission intensities would decrease due to the reduction in the number of Eu ions available for excitation/emission, however, the emission intensities are comparable. For example, the Eu concentration for the best CG Eu sample grown with Eu(DPM)_3_ (oxygen containing source) was ~6 × 10^19^ cm^−3^, while in the center of the 3nm DS samples it is ~4 × 10^19^ cm^−3^. Therefore, the 10:3 DS sample only has ~25% the Eu content of the CG sample, but 70% the integrated PL emission intensity. Thus, the emission intensity is not negatively impacted by the reduced Eu content, however, the reason for this is still under investigation.

To gain further insight into the structural properties of the overall Eu incorporation, the CEES technique was performed on two DS samples with GaN:Eu layer thicknesses of 3 nm and 10 nm. Results hereof are shown in Fig. 3c,d, respectively. For the sample with 3nm thick GaN:Eu layers, the CEES spectra reveal relatively sharp transitions in both excitation and emission (Fig. 3c). When the thickness of the GaN:Eu layer was raised to 10 nm, significant broadening and inhomogeneity is observed (Fig. 3d). This broadening is analogous to what was observed between CG GaN:Eu samples with and without oxygen co-doping (Fig. 2c,d). It should be noted, however, that no Eu precipitation was observed on the sample surface for any of the DS samples, thus all of the Eu atoms were stably incorporated into these materials.

Atom probe tomography (APT) has been utilized to investigate the doping profiles of the delta structures and quantify the extent of Eu diffusion between the Eu-doped layers. APT is an ideal technique for this type of analysis because of its ability to spatially resolve features at the nm-scale with a very high sensitivity due to low background levels. APT is a time of flight mass spectrometry technique in which a material is evaporated one ion at a time (See Methods).

**Figure 4:**
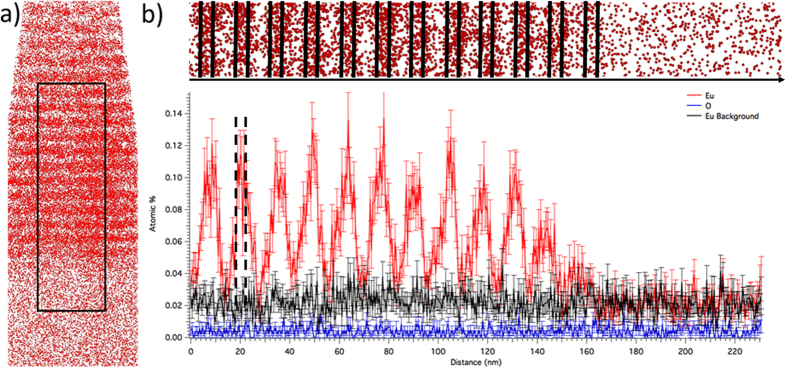
(**a**) A reconstructed APT image showing the Eu distribution of the DS samples with alternating 10 nm GaN layers and 4nm GaN:Eu layers. (**b**) Zoomed in view of the black box from Fig. 3a aligned with a plot of atomic percentage of Eu and O as a function of space. The background signal of Eu is also indicated for reference.

A reconstructed APT image of a DS sample with 4nm thick GaN:Eu layers is shown in Fig. 4a, and it is clear that there are in fact layers with a high Eu concentration separated by areas with a lower concentration, confirming that the DS is preserved during growth. Figure 4b is a 1D line profile in the z direction of the APT data shown in Fig. 3a showing O and Eu ions, including the Eu background. The Eu background is significantly higher than that for the O because the Eu^2+^ mass peaks (~75.5 and 76.5 Da) are detected slightly after the Ga^1+^ mass peaks (~69 and 71 Da), which have rather long tails due to material cooling after the initial laser pulse. The vertical black lines indicate the theoretical barriers of the layers, as expected from the growth procedure. It is clear that the Eu doping profile does not have abrupt interfaces because Eu diffuses several nms on either side of the 4nm intentionally doped regions. Therefore, much of the expected 10nm undoped layer is actually doped with Eu to concentrations above the APT detection limit. Despite this, there are regions between the doped layers that are absent of the Eu dopant, at least within the detection limit of APT. The O content is calculated to be ~0.005% throughout the entire dataset, which is significantly higher than the concentration determined by SIMS for the CG sample of a similar Eu flow rate (~0.00025%). Several factors can cause this discrepancy in the O content, such as residual O in the atom probe analysis chamber (~1e^−11^ Torr) that can field evaporate off the surface of the GaN. While a precise quantitative assessment of the O content is difficult, the content was monitored throughout the Eu doped layers to determine if higher concentrations of O were present in the presence of Eu. The 1D concentration profile does not show any deviation of the O content throughout the entire material, which will become important when discussing how the O/Eu ratio changes throughout the DS sample due to Eu diffusion.

The maximum Eu concentration in the center of the doped layer is on the order of ~8 × 10^19^ cm^−3^ or 0.1%, which is similar to the CG samples. Outside of the intentionally Eu doped region (indicated by vertical dotted black lines in Fig. 4b), there is significant Eu diffusion where the O to Eu ratio increases as the Eu concentration decreases. The increasing O/Eu ratio in the diffusion regions allows for the Eu ions to be properly incorporated, such that the Eu surface precipitation does not occur, and “good” optical properties are realized for the ≤4 nm Eu doped DS. However, the O/Eu ratio decreases to a value comparable to that of a CG sample without intentional O_2_ flow inside the well (~0.5%). This is based on the O content calculated by SIMS, and evidence from the APT dataset that the O content is constant.

APT was also performed on the 10:10 DS sample to compare the extent of Eu diffusion when the layer thickness is greatly increased, and to gain insight on the broadening of the CEES spectra (See Supplementary Figure 4). The Eu diffusion between the doped layers is nearly identical to the 10:4 sample, however, a much higher concentration of Eu remains in the well (~1.5×), which is spread through the entire 10 nm Eu doped layers, meaning that the O/Eu ratio is ~0.5 times the value for the 10:4 sample inside the well. This can easily be explained by the fact that there are more Eu ions in the 10 nm well than the 4 nm well, while approximately the same number of Eu ions diffuse into the 10 nm undoped layers for both samples. Therefore, a higher percentage of the Eu ions are withheld inside the 10 nm wells for the 10:10 vs. the 10:4 sample, where the O/Eu ratio is not ideal. Thus, the total number of native oxygen atoms in the 10 nm undoped layers are not sufficient to stabilize a majority of the Eu ions contained within the 10 nm layers. On the other hand, 10 nm of undoped GaN between 4 nm Eu layers does contain enough native O to stabilize a majority of Eu ions contained within the 4 nm layers. This is reflected in the CEES data. Overall, the Eu doped well thickness is optimized when there are a maximum number of Eu ions without sacrificing the optical properties due to a non-ideal O/Eu concentration, which enables the maximum number of emitting Eu ions to be incorporated into the material.

## Discussion

Our study reveals that oxygen clearly plays a critical role on the incorporation of Eu into GaN, and the optical properties of the resulting Eu centers. If substantial oxygen is not provided to the GaN:Eu layers, the Eu incorporation can become inhomogeneous, with many of the Eu atoms not located on a Ga site, and some even precipitating out of the crystal and onto the sample surface. The large areas of broadening in the CEES map of CG samples grown without additional oxygen (Fig. 2c) indicate that there are multiple “variations” of some of the incorporation centers, which differ in their local structure, as many of the Eu ions either sit off-center or at interstitials. Once oxygen is added, no Eu surface precipitation is observed, the RBS indicates that almost all of the Eu are on Ga sites, and the CEES become sharper and more uniform.

This observed dependence on oxygen would be quite unfavorable for the future proliferation of this material, however, for the thin (≤4 nm) GaN:Eu layers in the DS samples, it was observed that intentional oxygen co-doping was no longer required to attain acceptable structural and optical properties. The only O incorporated into these samples is that which is always present in OMVPE grown GaN. The concentration of this oxygen is over two orders of magnitude lower than those found in the samples grown with the O containing Eu(DPM)_3_ precursor, rendering the material compatible with current GaN-based devices.

These results are elucidated by the doping profiles acquired by APT, where spatially resolved diffusion of Eu atoms at the interfaces between the layers is observed. This Eu diffusion was expected from the precipitation seen on the surface of the CG sample when external O_2_ was not supplied. The ambient oxygen level in the 10:4 DS sample is observed to remain constant through the samples, thus at the edges of the diffusion regions, where the Eu concentration is very low, the O/Eu ratio can become larger than that measured for intentionally O co-doped CG samples, as determined by SIMS. As a result, the diffusion subsides and the resulting PL spectra are sharp. Thus, it is concluded that the majority of the Eu ions have “properly” incorporated themselves in the material, such that they are capable of emitting sharp and bright spectral lines.

On the other hand, once the thickness of the GaN:Eu layer reaches 10nm (i.e. the undoped layer thickness), approximately the same amount of Eu diffusion occurs between the Eu doped wells. However, a majority of the Eu ions are contained within the 10 nm doped well, and the total Eu concentration in this well is higher for the 10:10 vs. the 10:4 sample. Therefore, a majority of the Eu ions are subject to a concentration of available oxygen that is too low to facilitate a “favorable” growth environment for the 10:10 samples, and the emission broadening becomes comparable to the CG sample that is not co-doped with oxygen. Overall, for samples where the Eu doped layers are ≤4 nm, the CEES and APT data suggest that a sufficient majority of Eu find themselves in an environment that is sufficiently rich in O to stabilize their incorporation.

In summary, we have demonstrated that the O concentration in GaN:Eu materials can be reduced to a device-compatible level. It was concluded that periodic optimization of the concentration ratio between the normally occurring oxygen found in GaN and the Eu ions resulted in uniform Eu incorporation, without sacrificing emission intensity. These results appear to coincide with observations in other RE-doped GaN materials. Adoption of the methods discussed in this article could have a profound influence on the future optimization of these systems as well as GaN:Eu.

## Methods

### Sample fabrication

All continuous growth samples were grown on (0001) sapphire substrates by OMVPE. The samples are grown with a 30 nm low temperature GaN buffer layer, followed by a 2μm thick undoped GaN layer, and then a 300 nm GaN:Eu layer with a 10 nm thick capping layer (see Fig. 1a for a schematic of the structure). For GaN growth, the gallium and nitrogen sources were TMGa and ammonia, respectively. For comparison, Eu(DPM)_3_ and EuCp^pm^_2_ were both used as Eu precursors. To determine the effects of intentional oxygen co-doping, Ar diluted O_2_ gas was supplied during growth with flow rates of 100, 200 and 400 slm. To verify that there was no overall dependence on the particular oxygen source, NO gas was also used with flow rates of 200, 400 and 600 slm. The growth temperature and reactor pressure were 1030 °C and 100 kPa, respectively.

The delta structure samples were grown under similar conditions as the continuous growth samples, but with only the EuCp^pm^_2_ precursor. These samples are also grown with a 30 nm low temperature GaN buffer layer, followed by a 2 μm thick undoped GaN layer, and then 40 pairs of alternating GaN and GaN:Eu layers, where the GaN:Eu layers varied in thickness from 1 nm to 10 nm (see Fig. 2a for a schematic of the structure). The samples shown in the inset of Fig. 2b were grown at 960 °C to allow for maximum O incorporation in the un-doped layers.

### Optical measurements

To distinguish each emission center, and identify variations in local structures of these centers, combined excitation-emission spectroscopy (CEES) was used^46^,^47^. The ^7^F_0_-^5^D_0_ transition of Eu^3+^ occurs between singlet states and involves no fine-structure splitting, which means that each incorporation site should only be excited at one excitation energy. However, small variations in the local structure or strain environment around each center can still be detected in the CEES maps by the appearance of inhomogeneous line broadening or fluorescence line narrowing.

Eight distinct Eu incorporation centers have been identified in OMVPE grown GaN:Eu^47^. Two of these centers, referred to as Eu1 and Eu2, are of considerable importance. Eu1 is the majority center in GaN:Eu, comprising over 75% of the Eu incorporation, while PL emission from Eu2 is the most prominent under excitation above the bandgap, despite its lower abundance (≈4%). Additional details about the CEES techniques as well as the incorporation centers and their optical properties in GaN, can be found in other reports^43^,^46^,^47^.

For excitation above the bandgap (indirect), a 325 nm He-Cd laser was used. All emission spectra were collected using a 0.5 m spectrometer with a charge couple device (CCD). For low temperature measurements (≈10K), the samples were mounted to a closed-cycle liquid helium cryostat.

### Atom probe tomography

Sample preparation was completed with the wedge lift-out technique using an FEI Nova 800 FIB and mounted on Si microtip substrates supplied by CAMECA. 30 kV Ga+ annular milling was followed by a 5 kV ion cleaning step to remove the Pt cap and Ga ion-induced surface damage, and to obtain a resulting initial needle radius below 50 nm. The GaN needles were run in a CAMECA LEAP 4000X-HR with a 50 kHZ pulse frequency, 2% detection rate, ~0.2 pJ laser energy resulting in a Ga+/Ga++ peak ratio of ~50, and a base temperature of 25 K. The low pulse rate was used to limit the effects of DC heating, which results in a larger signal to noise ratio than higher pulse rates. A slightly higher than normal laser power was also used to increase the signal to noise ratio. Although the Ga/N ratio using these conditions was ~60/40 rather than 50/50, the measured Eu and O content was not significantly different compared to data acquired using a lower laser energy (0.04 pJ) and a Ga/N ratio of 50/50. The detector event histogram showed even evaporation of the GaN needle. The tip profile reconstruction was used, such that the total thickness of each pair of layers is measured to be ~14 nm, as expected.

## Additional Information

**How to cite this article**: Mitchell, B. *et al.* Utilization of native oxygen in Eu(RE)-doped GaN for enabling device compatibility in optoelectronic applications. *Sci. Rep.* **6**, 18808; doi: 10.1038/srep18808 (2016).

## Supplementary Material

Supplementary Information
