# Supplementary material for: Utilization of native oxygen in Eu(RE)-doped GaN for enabling device compatibility in optoelectronic applications
Source: Sci Rep. 2016 Jan 4;6:18808. doi: 10.1038/srep18808 (PMC4698738; doi:10.1038/srep18808)
Supplement: Supplementary Information [file srep18808-s1.doc]

**Utilization of native oxygen in Eu(RE)-doped GaN for enabling device compatibility in optoelectronic applications**

B. Mitchell^1*^, D. Timmerman^2^, J. Poplawsky^3^, W. Zhu^2^, D. Lee^2^, R. Wakamatsu^2^, J. Takatsu^2^, M. Matsuda^2^, W. Guo^3^, K. Lorenz^4^, E. Alves^4^, A. Koizumi^2^, V. Dierolf^5^, and Y. Fujiwara^2^

^1^Department of Physics and Astronomy, University of Mt. Union, 1972 Clark Ave, Alliance, OH, 44601, USA

^2^Division of Materials and Manufacturing Science, Graduate School of Engineering, Osaka University, Suita, Osaka 565-0871, Japan

^3^Center for Nanophase Materials Sciences, Oak Ridge National Laboratory, Oak Ridge, TN 37831, USA

^4^Instituto Superior Técnico, Campus Tecnológico e Nuclear, Estrada Nacional 10, P-2695-066 Bobadela LRS, Portugal

^5^Department of Physics and Astronomy, Lehigh University, 16 Memorial Dr. E, Bethlehem, PA, 18015, USA

**SUPPLEMENTARY INFORMATION**

**Section 1: Rutherford Backscattering Data Extended Explanation**

Rutherford backscattering/channeling (RBS/C) measurements were performed on samples with and without oxygen co-doping to explore the effect of oxygen on the lattice location of the Eu ions. Figure 1 in the manuscript shows the angular RBS/C scans across the <0001> and <10-11> axes for the two samples. In the sample with oxygen co-doping, the near identical overlap and width of the scans for Eu and Ga shows that Eu is incorporated almost entirely on substitutional Ga-sites. This result is identical to the case of sample grown with Eu(DPM)_3_. On the contrary, about 30% of the Eu ions are located in random positions in the sample without oxygen co-doping. The remaining 70% seem to be slightly displaced from the substitutional Ga-sites, which is inferred from the slightly narrower shape of the Eu-scans as compared to the Ga-scans. Consequently, oxygen plays a significant role in the uniform substitution of Eu ions onto Ga-sites. We verified that oxygen alone was responsible for this effect by also using NO gas as a source for oxygen co-doping. There was no noticeable difference in the resulting structural properties as compared to using Ar diluted O_2_.

**Section 2: Eu Precipitation in CG Samples**

The RBS data indicates that the Eu are not incorporated the way that was observed in samples grown with Eu(DPM)_3_. Looking at the surface, a clear precipitation is observed for samples grown with the oxygen-free Eu source when no O_2_ is supplied in Supplementary Figure 1 (a). Once O_2_ is supplied, keeping the Eu flow rate the same, this precipitation is no longer observed [Supplementary Figure 1 (b)]. However, when the Eu flow rate is substantially increased, keeping the O_2_ flow rate the same, the precipitation returns. This precipitation is due to Eu atoms that have precipitated out of the sample and stabilized on the surface as Eu-N, which was confirmed by an analysis of transmission electron microscopy diffraction patterns.

It is evident that there is Eu diffusion when sufficient O_2_ is not supplied in CG samples, some of the Eu stabilize in the sample as interstitials as seen from the RBS data, while others diffuse out of the sample and are observed on the sample surface. This precipitation is not observed for the DS samples explored in this study [Supplementary Figure 1 (c)]. This can be understood from the doping profiles attained by APT, where even for the 10:10 sample the diffusion is significantly hindered by the undoped layers.


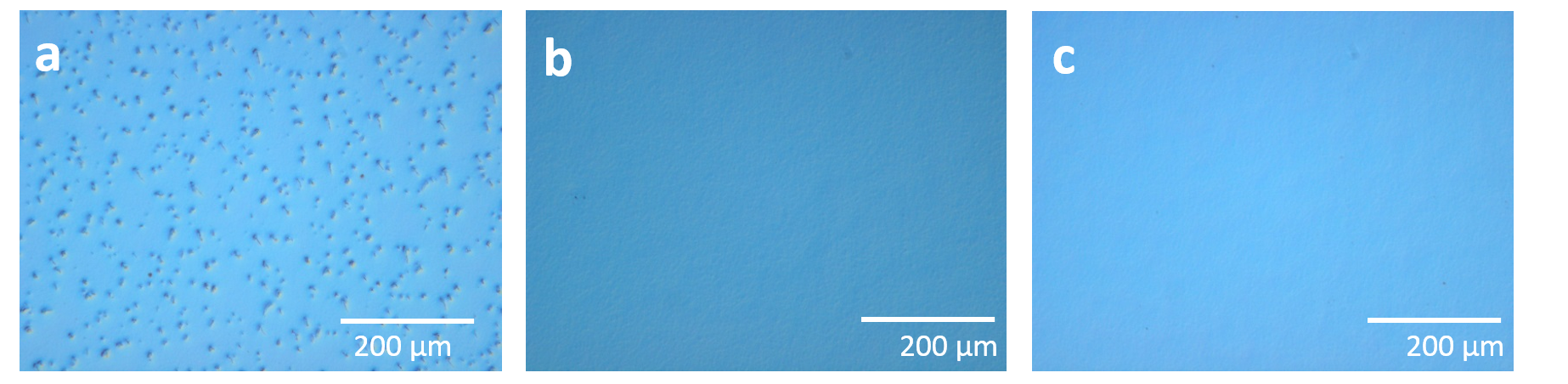


**Supplementary Figure 1:** Images of sample surfaces for CG GaN:Eu samples without O_2_ supplied (a), and with O_2_ supplied (b). For the sample where O_2_ is not supplied, Eu precipitation is observed, however, it is no longer present once O_2_ is supplied. (c) The precipitation is also not observed for the DS samples, even though O_2_ is not supplied.

**Section 3: Photoluminescence Data Extended**

We have shown that the absence of oxygen results in a substantial structural variation of the incorporated Eu ions, which should significantly influence the optical properties of the system. Thus, we explored the influence of oxygen on the shape and intensity of photoluminescence (PL) emission under the indirect excitation of the Eu ion. The PL emission spectra for GaN:Eu samples grown with the oxygen free Eu source and with increasing oxygen flow rates are shown in Fig. 1b of the manuscript. When no additional oxygen is supplied to the sample, the resulting PL spectrum is very broad and relatively weak. The emission peaks in the spectra become sharp and increase in intensity when oxygen is added and the flow rate is increased. The relative heights of these peaks also change, which indicates that the oxygen may be preferentially benefiting certain Eu ions. These sharp emission peaks are characteristic of samples grown with Eu(DPM)_3_.

Furthermore, indirect excitation requires the trapping of e-h pairs and the subsequent energy transfer to the Eu ions after their recombination. For this to occur, defects local to the Eu ion are necessary to localize the e-h pair to the Eu ion. The effect of co-doping oxygen on this localization, for various oxygen concentrations, can also be seen in Fig. 1b of the main article. As the oxygen concentration is increased, there is a substantial increase in the integrated emission intensity, particularly for the third peak at 1.996 eV, which is associated with the Eu incorporation configuration referred to as Eu2 [refer to methodology]. This implies that some Eu incorporation centers may benefit more from the addition of oxygen than others.

To further explore the modifications of the local structure of the Eu ions in the samples due to the presence of oxygen, the technique of combined excitation emission spectroscopy (CEES) was utilized (refer to methodology). Supplementary Figure 2 shows the CEES results for the samples (a) doped with Eu(DPM)_3_, (b) doped with EuCp^pm^_2_ only, (c) co-doped with EuCp^pm^_2_ and Ar diluted O_2_, respectively. The samples grown with Eu(DPM)_3_ exhibit very sharp excitation and emission peaks, and numerous individual incorporation centers can be identified [Supplementary Figure 2 (a)]. When the oxygen-free Eu source (EuCp^pm^_2_) was used alone, only a few centers can still be discretely identified [Supplementary Figure 2 (b)]. The rest of the CEES map shows large areas of broadening and tilting in both the excitation and emission. This is a result of the inhomogeneity of the local defect and strain environments around the Eu ions for each of the incorporation centers. Once additional oxygen is introduced during growth, the sharp transitions are recovered [Supplementary Figure 2 (c)]. This indicates that oxygen plays a large role in the homogeneity of the Eu incorporation into the GaN host. Furthermore, it was found that samples without oxygen co-doping had a significant amount of Eu precipitation on the surface. This precipitation was no longer observed once the samples were co-doped with oxygen. Therefore, in addition to the distortion of the local Eu structures, many of the Eu ions did not remain in the sample, but precipitated out of the crystal.

**
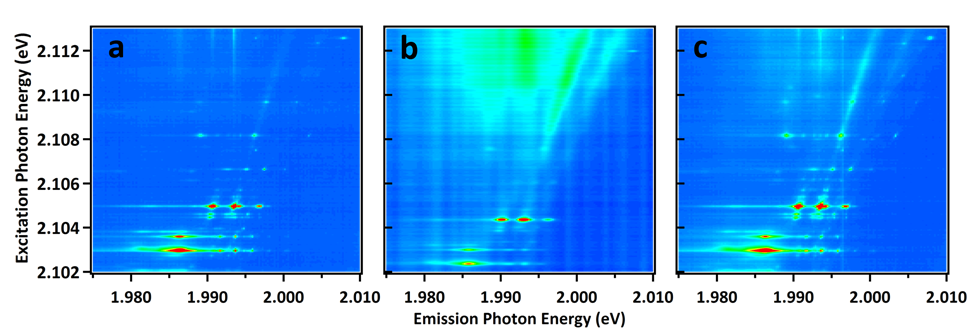
**

**Supplementary Figure 2:** CEES Spectra for GaN:Eu samples grown with two different Eu sources and with/without oxygen co-doping. The samples were (a) doped with Eu(DPM)_3_, (b) doped with EuCp^pm^_2_ only, (c) co-doped with EuCp^pm^_2_ and O_2_, respectively.

**Section 4: SIMS Confirmation of Average Oxygen and Eu Concentrations**

To gain insight on the role of Eu on the incorporation of oxygen, SIMS measurements were performed on samples where the flux of the EuCp^pm^_2_ was varied while the O_2_ flux was fixed at 5 × 10^-4^ sccm. The EuCp^pm^_2_ flux dependence on the concentrations of Eu and O are shown in Supplementary Figure 3. As expected, when the Eu flux is increased, the concentration of Eu increases. Interestingly, despite the fixed flux of the O source, the concentration of oxygen increases proportionally to the increase in Eu concentration. In fact, the ratio of O to Eu remains fixed around 2.5% for all Eu source fluxes. Therefore, the incorporation of O into the crystal is dependent on the Eu concentration. If there are more Eu ions available, more O will be incorporated into the sample. When additional oxygen is not supplied, this percentage cannot be met because the concentration of native O in GaN is over two orders of magnitude less than the Eu concentration required to produce adequate luminescence intensity.


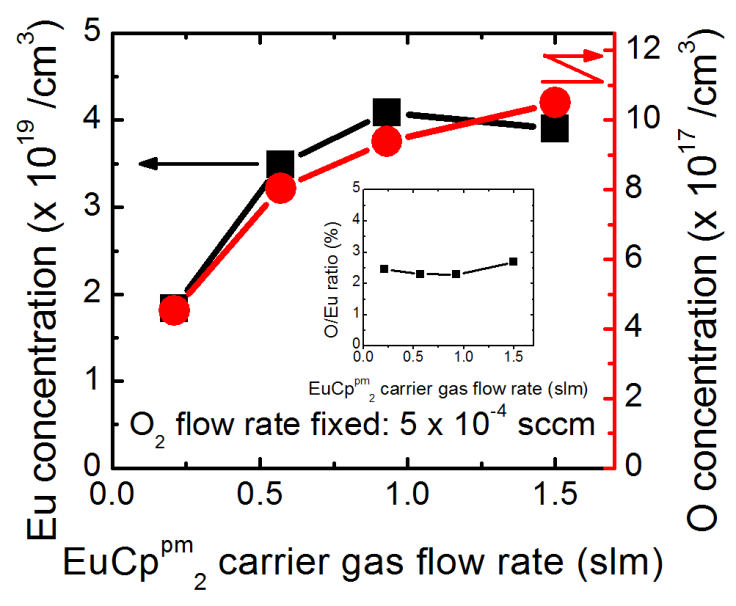


**Supplementary Figure 3:** SIMS results for GaN:Eu samples with varying Eu source flow rates, with a fixed O flow rate.

**Section 5: APT Data Extended**

The Eu distribution of the DS sample with alternating 10nm GaN layers and 10nm GaN:Eu layers (10:10 sample) was also observed with APT (Supplementary Figure 4). We obtained the 1D compositional profiles along regions of Eu doped quantum wells [Supplementary Figure 4 (b)]. For comparison, two wells from the 1D line profile data from Figure 4 of the manuscript (10:4 sample) are overlaid with the 10:10 sample, which show that the Eu diffusion into the undoped layers is relatively the same for both samples. However, a higher Eu concentration remains in the 10 nm doped wells compared to the 4 nm doped wells. This can be interpreted by the same amount of Eu diffusion in between the wells while the 10:10 sample starts with more Eu ions in the 10 nm doped wells.


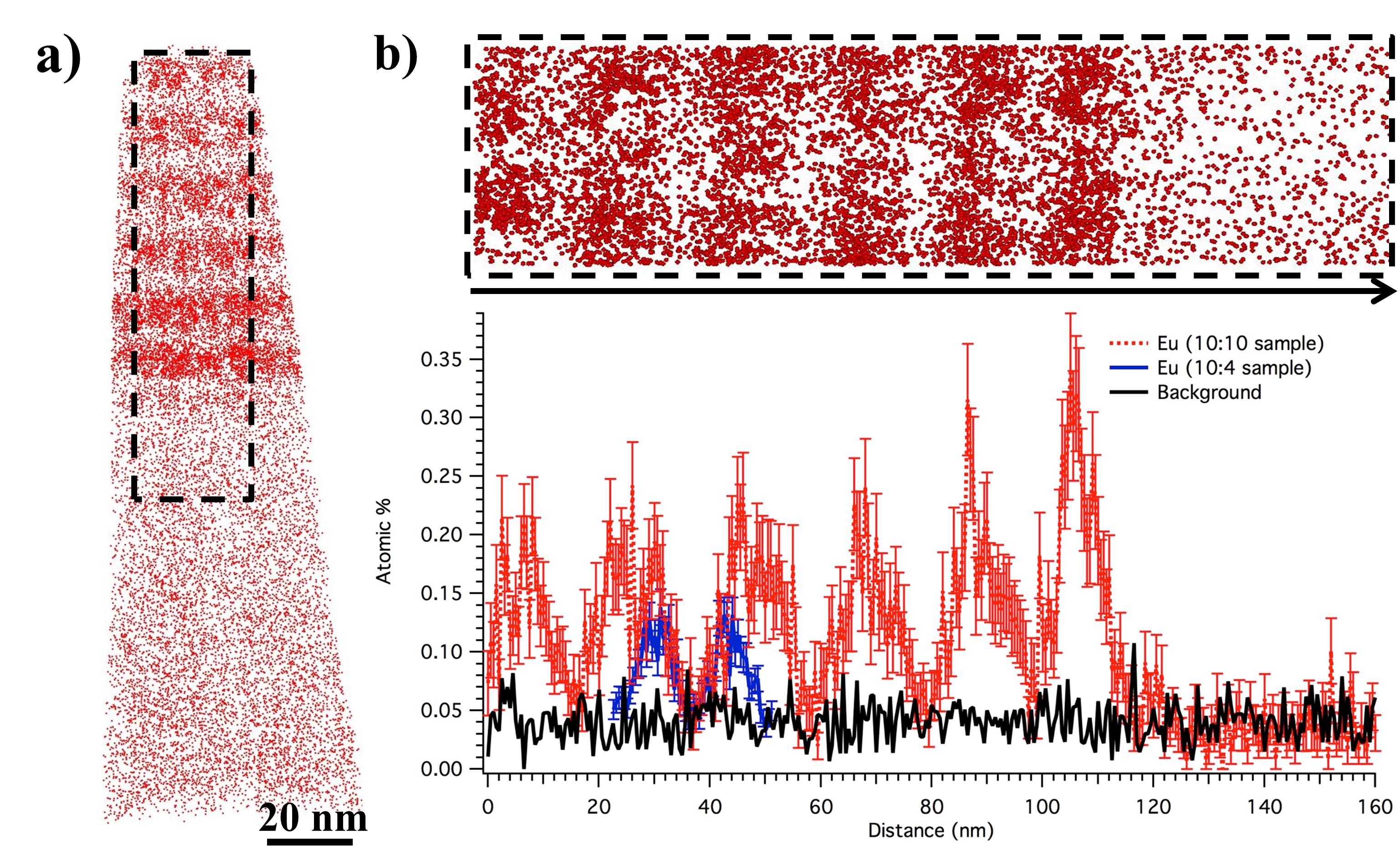


**Supplementary Figure 4:** (a) A reconstructed APT image showing the Eu distribution of the DS sample with alternating 10nm GaN layers and 10nm GaN:Eu layers.  (b) Magnification of sub-regions taken from (a). Also shown is the 1D line profile along the arrow. The background signal of Eu ions and two wells from the DS 10:4 sample shown in Figure 3 are also included for reference.
